# Clinical analysis of patients with skeletal metastasis of lung cancer

**DOI:** 10.1186/s12885-019-5534-3

**Published:** 2019-04-03

**Authors:** Yong Jin Cho, Yung Min Cho, Sung Hyun Kim, Kyoo-Ho Shin, Sung-Taek Jung, Hyo Song Kim

**Affiliations:** 1 Department of Orthopedic Surgery, Chosun University College of Medicine, Gwangju, South Korea; 2 Department of Orthopedic Surgery, Yonsei University College of Medicine, Seoul, South Korea; 3 Department of Orthopedic Surgery, Chonnam National University College of Medicine, Gwangju, South Korea; 4 Division of Medical Oncology, Department of Internal Medicine, Yonsei University College of Medicine, 134 Shinchondong, Seodaemun-gu, Seoul, 120-752 South Korea

**Keywords:** Lung neoplasms, Multivariate analysis, Bone neoplasms

## Abstract

**Background:**

Many factors influence bone metastases of lung cancer, and several studies report about survival of skeletal metastasis. However, few studies have focused on identifying the prognostic factors for skeletal metastasis of lung cancer, especially following orthopedic surgery. We conducted a retrospective analysis of the clinical characteristics of skeletal metastasis from lung cancer and discuss the prognostic factors.

**Methods:**

We performed a medical record review of 202 patients who were diagnosed with skeletal metastasis from lung cancer. Adenocarcinoma was found in 116 patients (57.4%), squamous cell carcinoma in 29 (14.4%), small-cell lung cancer (SCLC) in 37 (18.7%), and large-cell carcinoma and other types of cancer in 20 patients (9.9%). Orthopedic surgery for skeletal metastasis was performed in 41 patients (20.3%).

**Results:**

Lung cancer survival was 12.1 months. After diagnosis of lung cancer, skeletal metastasis was found at a mean of 2.5 months, and skeletal metastasis survival was 9.8 months. Lung cancer survival in patients younger than 60 years was 13.8 months, and lung cancer survival in patients 60 years or older was 10.8 months (*p* = 0.009). Skeletal metastasis survival in patients younger than 60 years was 11.0 months, and skeletal metastasis survival in patients 60 years or older was 8.8 months (*p* = 0.002). Mean skeletal metastasis survival with surgery was 12.6 months and without surgery was 9.1 months (*p* < 0.000). In the multivariate analysis of lung cancer survival, age under 60 years [HR (95% CI) 1.549 (1.122–2.139), *p* = 0.008], non-small cell lung cancer pathology type [HR (95% CI) 1.711 (1.157–2.532), *p* = 0.008], chemotherapy for skeletal metastasis [HR (95% CI) 8.064 (3.981–16.332), *p* < 0.000], and radiation therapy for skeletal metastasis [HR (95% CI) 1.791 (1.170–2.742), *p* = 0.007] were significant, independent, good prognostic factors. In the multivariate analysis of skeletal metastasis survival, age under 60 years [HR (95% CI) 1.549 (1.124–2.134), *p* = 0.007], non-small cell lung cancer pathology type [HR (95% CI) 2.045 (1.373–3.047), *p* < 0.000], chemotherapy for skeletal metastasis [HR (95% CI) 7.121 (3.542–14.317), *p* < 0.000], and orthopedic surgical treatment for skeletal metastasis [HR (95% CI) 1.710 (1.148–2.547), *p* = 0.008] were significant, independent, good prognostic factors.

**Conclusions:**

Patients who survived longer were less than 60 years old, received chemotherapy as treatment for skeletal metastasis, had NSCLC rather than SCLC, and underwent orthopedic surgery for skeletal metastasis.

## Background

Lung cancer is the leading cause of cancer-related deaths worldwide, accounting for almost 20% of cancer-related fatalities [1, 2]. The incidence of lung cancer is about 53.6/100,000 people every year, and mortality is 45.6/100,000 every year [3]. The skeletal system is one of the most common distal metastatic sites in patients with lung cancer, and 30–40% of those with advanced lung cancer develop skeletal metastases [4, 5]. Skeletal metastasis involves significant morbidity, metabolic disorders such as hypercalcemia, pathologic fractures, and spinal cord compression. These disease sequelae entail a reduction in quality of life and require costly treatments that have limited impact on overall survival. The importance of metastasis from lung cancer has been overlooked in patients with advanced lung cancer because the mean survival is less than 6 months [6]. The recent development of remarkable chemotherapy and supportive therapies has caused the average survival time in patients with lung cancer to dramatically increase, and interest in skeletal metastasis of lung cancer has increased. Pathologic fractures, or skeletal-related events (SREs), following the skeletal metastasis of lung cancer can substantially reduce quality of life and increase economic burden. Some reports have stated that the incidence of SREs are reduced with use of medications, such as bisphosphonates and denosumab, for lung cancer that has metastasized to the limbs of the skeletal system [7–10]. Traditionally, orthopedic surgery, such as open reduction internal fixation, has been an important treatment method. Many factors influence bone metastases of lung cancer, including age, sex, pathologic type, number of primary lesions, number of bone metastases, treatment regimens, and serum markers [11]. However, few studies have focused on identifying the prognostic factors for skeletal metastasis from lung cancer, especially following orthopedic surgery. Therefore, we conducted a retrospective analysis of the clinical characteristics of skeletal metastasis from lung cancer and discuss the prognostic factors.

## Methods

### Participants

From January 1, 2005 to December 31, 2015, a total 329 patients with lung cancer who were diagnosed with skeletal metastatic disease were recruited from three institutions. For the purpose of analysis, the following exclusion criteria were applied: 1) patients who had only spinal metastasis without appendix bone metastasis (*n* = 82) and 2) patients who had undergone an operation on the small bones of the hands or feet (*n* = 3). Of the remaining 244 patients, 42 patients with less than 24 months of follow up were excluded, which left us with 202 patients for medical record review.

**Fig. 1:**
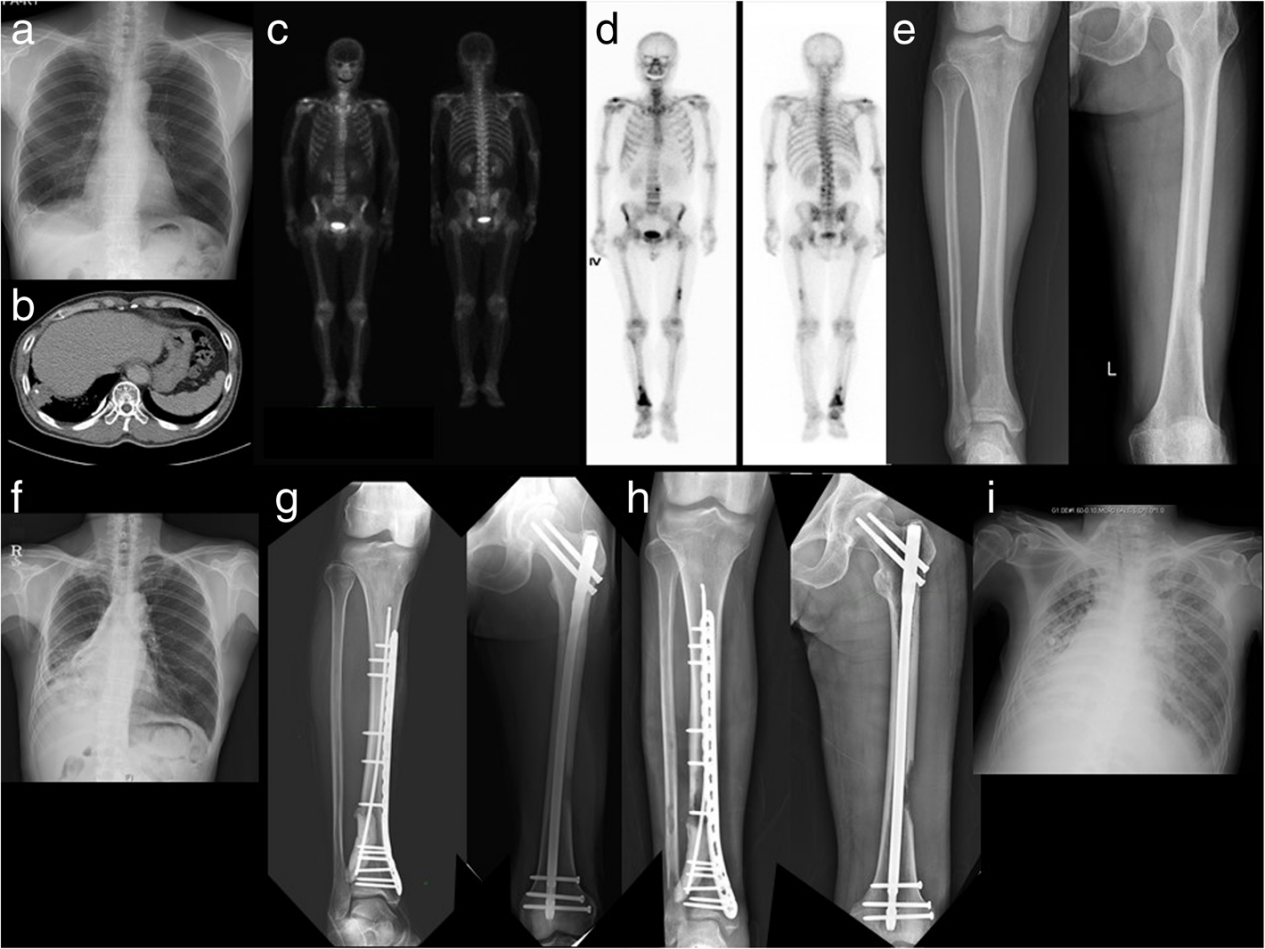
A 66-year-old man who visited our hospital for hemoptysis. **a** Chest X-ray showed right costo-phrenic angle blurring. **b** Chest CT scan showed a 4 cm-sized mass. Squamous cell carcinoma was diagnosed by bronchoscopic biopsy. **c** At the time of diagnosis, the stage was T3N2M0. **d** At follow-up, WBBS was performed at 20 months of diagnosis, and hot-uptake was observed at the 2nd Lumbar body, left distal femur diaphysis and right distal tibia diaphysis. **e** The osteolytic lesions are observed in the lateral cortex of the left femur diaphysis and lateral cortex of right tibia diaphysis, suggesting skeletal metastases. **f** In chest X-ray, the haziness was increased at the right mid and lower lung field. **g** Curettage, flexible intramedullary nailing, plate fixation and bone cementing were performed on the right distal tibia diaphysis metastasis. Interlocking intramedullary nailing was performed on the left femur distal diaphysis metastasis. Additional postoperative radiation therapy was performed, and chemotherapy was continued. **h** Ten months after surgery, there was a slight increase in the size of the osteolytic lesions around the surgical sites, but full weight bearing without pain was possible. **i** The patient expired from pneumonia associated with lung cancer

Of the total 202 patients, 126 (62.4%) were male and 76 (37.6%) were female. The mean age was 61.2 years (range: 29–85 years). Adenocarcinoma was found in 116 patients (57.4%), squamous cell carcinoma in 29 (14.4%), small-cell lung cancer (SCLC) in 37 (18.7%), and large-cell carcinoma and other types of cancer in 20 patients (9.9%). A total 114 patients (56.4%) had a history of smoking at the time of diagnosis, and 88 (43.6%) had no smoking history. Hypertension was diagnosed in 50 cases (24.8%), and diabetic mellitus was 26 cases (12.9%), chronic renal failure was 3 cases (1.5%). Chemotherapy was administered in 192 cases (95.0%) for skeletal metastasis from lung cancer, and radiation therapy was used in 174 cases (86.1%) (Table 1). We reviewed plain radiography, magnetic resonance imaging (MRI), computed tomography (CT), whole body bone scan (WBBS), and positron emission tomography–computed tomography (PET-CT). When the patient’s imaging results showed two or more positive findings, the patient was confirmed to have skeletal metastases. In 41 patients who underwent orthopedic surgery, pathologic examination of the metastatic site was performed. In all cases, metastatic lung cancer was reported. A typical example from diagnosis to progression, operation and the results was shown in Fig. 1. The patients were followed for 24 months in an open patients department visit and/or telephone interview. We analyzed the survival rate and prognostic factors of patients with lung cancer who had skeletal metastases (Table 1). Ethics approval was obtained from the Ethics Committee from all three participating hospitals.

**Table 1:** Demographic data of patients with skeletal metastasis from lung cancer

			Lung cancer survival		Skeletal metastasis survival	
	Patients, n (%)		Survival (months)	*p*-value	Survival (months)	*p*-value
Age, years						
< 60	88 (43.6%)		13.8	0.009	11.0	0.002
≥ 60	114 (56.4%)		10.8		8.8	
Sex						
Male	126 (62.4%)		12.2	0.850	9.5	0.327
Female	76 (37.6%)		11.9		10.2	
Pathology						
Adenocarcinoma	116 (57.4%)	NSCLC	12.6	0.080	10.4	0.000
Squamous cell carcinoma	29 (14.4%)					
Large cell and other type lung cancer	20 (9.9%)					
Small cell lung cancer	37 (18.3%)	SCLC	10.0		7.2	
Smoking						
Smoker	114 (56.4%)		12.0	0.968	9.4	0.216
Non-smoker	88 (43.6%)		12.1		10.3	
Hypertension						
No	152 (75.2%)		11.9	0.545	9.6	0.421
Yes	50 (24.8%)		12.7		10.2	
Diabetic Mellitus						
No	176 (87.1%)		11.9	0.400	9.6	0.332
Yes	26 (12.9%)		13.3		10.6	
Chronic renal failure						
No	199 (98.5%)		12.1	0.960	9.7	0.370
Yes	3 (1.5%)		12.3		12.3	
Chemotherapy						
Do	192 (95%)		12.6	0.000	10.1	0.000
Undo	10 (5%)		3.2		3.2	
Radiation therapy						
Do	174 (86.1%)		12.5	0.062	9.9	0.147
Undo	28 (13.9%)		9.5		8.5	
Orthopedic surgery						
Do	41 (20.3%)		14.1	0.079	12.6	0.000
Undo	161 (79.7%)		11.6		9.1

### Statistical analysis

The general characteristics and basic data of patients were summarized using descriptive statistics. The correlation between survival length after diagnosis of lung cancer, diagnosis of skeletal metastasis, and clinicopathologic variables was analyzed using an independent sample *t*-test. For survival analysis, lung cancer survival was defined as the time interval between the diagnosis of lung cancer and death or the last follow-up. Skeletal metastasis survival was defined as the time interval between the diagnosis of skeletal metastasis from lung cancer and death or the last follow-up. Lung cancer survival analyses and skeletal metastasis survival analyses were conducted according to the Kaplan-Meier method. Univariate and multivariate analyses were done using a proportional hazard regression model (cut-off *p* value of 0.05). A multivariate analysis included significant and non-redundant variables selected from the univariate analysis [12]. All statistical analyses were carried out using IBM SPSS version 18.0 (SPSS Inc., Chicago, Illinois, USA). Results were considered statistically significant if *p* < 0.05.

## Results

### Demographic data

A total of 128 patients (63.4%) were found to have multiple distant metastases, including skeletal metastases, at the time of lung cancer diagnosis, and 35 patients (17.3%) only had skeletal metastases at the time of lung cancer diagnosis. Epidermal growth factor receptor (EGFR) mutation was detected in 18 patients (8.9%) and not detected in 53 patients (26.2%); EGFR mutation tests were not performed in the remaining 131 patients (64.9%). There were 43 patients (21.3%) with one other distant organ metastasis, 78 (38.6%) with two, 52 (25.7%) with three, 22 (10.9%) with four, and six patients (3.0%) with five other distant organ metastases. One patient (0.5%) had more than six other distant organ metastases at the time of diagnosis of skeletal metastasis. Distant organ metastases included 20 cases involving the mediastinum, 69 of the liver, 177 involving the lymph nodes, 29 of the chest wall, 101 of the cranial cavity, 55 of the adrenal glands, and 28 cases involving the kidneys.

Lung cancer survival was 12.1 months (range: 1–44 months). After diagnosis of lung cancer, skeletal metastasis was found at a mean of 2.5 months (range: 0–27 months); other organ metastases were found at a mean of 2.9 months (range: 0–24 months) after diagnosis of lung cancer. Orthopedic surgical treatment was performed in 41 patients (20.3%). At the last follow-up, 166 patients (82.20%) had died; skeletal metastasis survival was 9.8 months (range: 1–24 months).

Among patients diagnosed with skeletal metastasis, patients younger than 60 years survived an average of 13.8 months, and those aged 60 years or older survived an average of 10.8 months after diagnosis of lung cancer (*p* = 0.009). After diagnosis of skeletal metastasis, patients younger than 60 years survived an average of 11.0 months, and those aged 60 years or older survived an average of 8.8 months (*p* = 0.002).

After the diagnosis of lung cancer, the average survival rate was 12.2 months in male patients and 11.9 months in female patients (*p* = 0.850). Males survived an average of 9.5 months and females survived an average of 10.2 months after skeletal metastasis diagnosis (*p* = 0.327). After a diagnosis of lung cancer, the 114 patients with a smoking history (56.4%) survived an average of 12.0 months, and the 88 patients with no smoking history survived an average of 12.1 months (*p* = 0.968). Patients with non-small cell lung cancer (NSCLC) survived an average of 12.6 months after diagnosis of lung cancer, and those with small-cell carcinoma survived an average of 10.0 months (*p* = 0.080). Patients with NSCLC survived an average of 10.4 months after diagnosis of skeletal metastasis, and those with small-cell carcinoma survived an average of 7.2 months (*p* = 0.000). We could not find any statistical significances in lung cancer survival and skeletal metastasis survival in patients with and without hypertension, diabetic mellitus, chronic renal failure (Table 1).

One hundred ninety-two patients (95%) underwent chemotherapy for skeletal metastasis, and their lung cancer survival was 12.6 months. Lung cancer survival in 10 patients (5%) without chemotherapy was 3.2 months (*p* < 0.000). The skeletal metastasis survival of patients who underwent chemotherapy for skeletal metastasis was 10.1 months, and the skeletal metastasis survival of patients who were not treated with chemotherapy was 3.2 months (*p* < 0.000). One hundred seventy-four patients (86.1%) underwent radiation therapy for skeletal metastasis, and their lung cancer survival was 12.5 months. Lung cancer survival in 28 patients (13.9%) without radiation therapy was 9.5 months (*p* = 0.062). The skeletal metastasis survival of patients who underwent radiation therapy for skeletal metastasis was 9.9 months, and the skeletal metastasis survival of patients who were not treated with radiation was 8.5 months (*p* = 0.147).

Forty-one patients (20.3%) underwent orthopedic surgery for pathologic fractures or impending fractures secondary to the underlying skeletal metastasis from lung cancer. The average age of patients with a diagnosis of lung cancer who underwent orthopedic surgery was 59.7 years; the average age of patients who did not have surgery was 61.4 years (*p* = 0.374). Mean lung cancer survival of patients who underwent orthopedic surgery was 14.1 months, and mean lung cancer survival was 11.6 months in patients without surgery (*p* = 0.079). Mean skeletal metastasis survival in patients who underwent surgery was 12.6 months; without surgery, mean survival after diagnosis of skeletal metastasis was 9.1 months (*p* < 0.000) (Table 1).

### Univariate and multivariate regression analysis of lung cancer survival

**Fig. 2:**
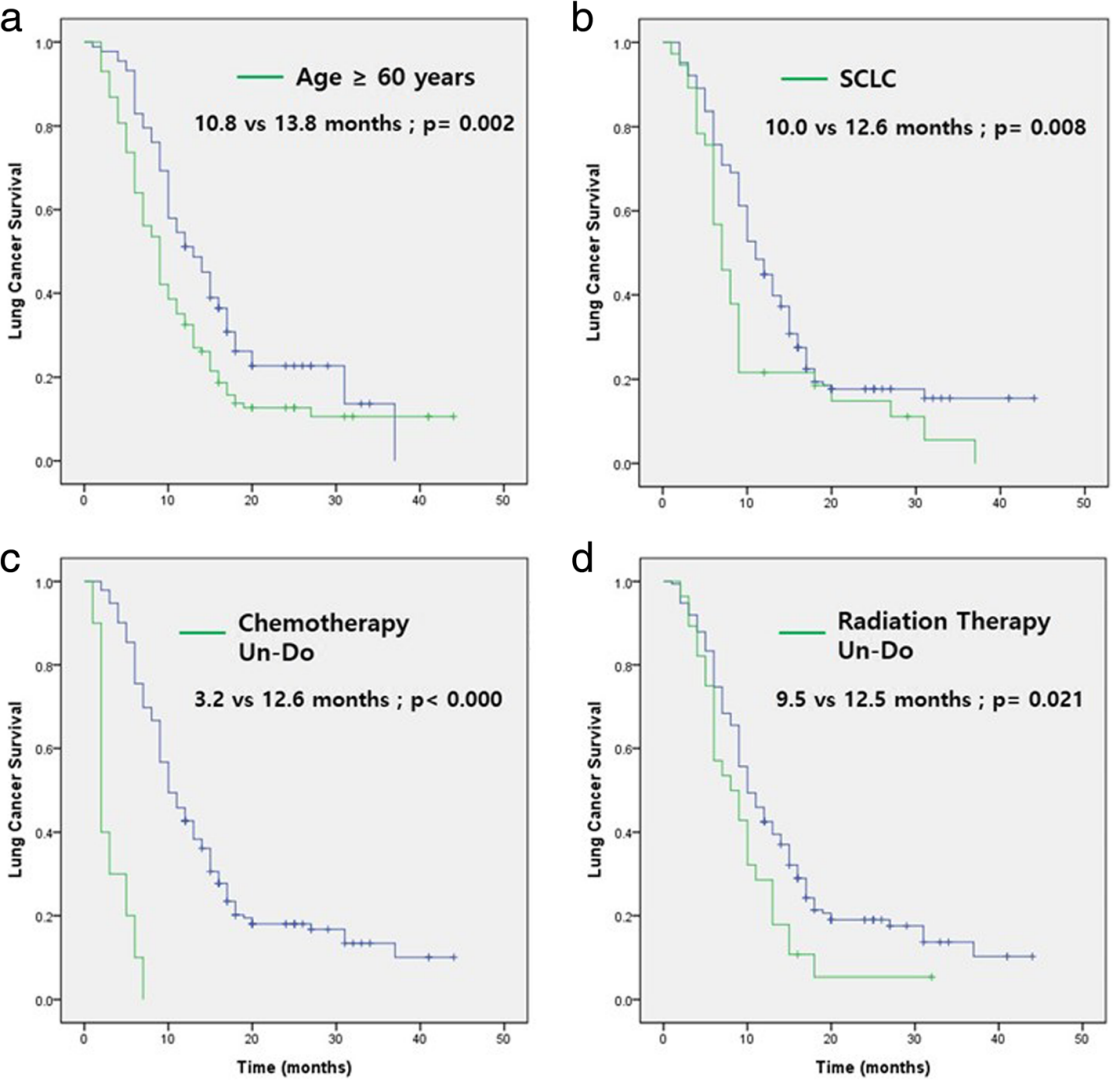
Kaplan-Meier plots of lung cancer survival according to **a**) age at diagnosis of lung cancer, **b**) pathologic type of lung cancer, **c**) whether chemotherapy was performed for skeletal metastasis, and **d**) whether radiation therapy was performed for skeletal metastasis. Median survivals are expressed in months. Statistical significance was assessed by the log-rank test

Lung cancer diagnosed under 60 years of age [HR (95% CI) 1.538 (1.159–2.161), *p* = 0.002], small cell lung cancer pathology type [HR (95% CI) 1.630 (1.114–2.385), *p* = 0.008], chemotherapy for skeletal metastasis [HR (95% CI) 9.827 (4.939–19.552), *p* < 0.000], and radiation therapy for skeletal metastasis [HR (95% CI) 1.597 (1.048–2.434), *p* = 0.021] were significantly associated with longer lung cancer survival (Fig. 2). In addition, the multivariate analysis performed to determine which factors were an independent prognostic factor revealed that age under 60 years at diagnosis [HR (95% CI) 1.549 (1.122–2.139), p = 0.008], small cell lung cancer pathology type [HR (95% CI) 1.711 (1.157–2.532), *p* = 0.007], chemotherapy for skeletal metastasis [HR (95% CI) 8.064 (3.981–16.332), *p* < 0.000], and radiation therapy for skeletal metastasis [HR (95% CI) 1.791 (1.170–2.742), *p* = 0.007] were significant independent prognostic factors (Table 2).

**Table 2:** Univariate and multivariate regression analysis of lung cancer survival

Variable	Univariate analysis			Multivariate analysis		
	HR	95% CI	*p*-value	HR	95% CI	*p*-value
Age (< 60 vs. ≥ 60)	1.538	1.159–2.161	0.002	1.549	1.122–2.139	0.008
Sex (male vs. female)	1.086	0.790–1.492	0.595			
Smoking (nonsmoker vs. smoker)	1.114	0.818–1.517	0.473			
Pathology (NSCLC vs. SCLC)	1.630	1.114–2.385	0.008	1.711	1.157–2.532	0.007
EGFR (positive vs. negative)	1.462	0.769–2.780	0.225			
Number of distant organ metastasis (only 1 vs. over 2)	1.177	0.812–1.705	0.367			
Chemotherapy (yes vs. no)	9.827	4.939–19.552	0.000	8.064	3.981–16.332	0.000
Radiation therapy (yes vs. no)	1.597	1.048–2.434	0.021	1.791	1.170–2.742	0.007
Orthopedic surgery (yes vs. no)	1.377	0.931–2.036	0.092			

### Univariate and multivariate regression analysis of skeletal metastasis survival

**Fig. 3:**
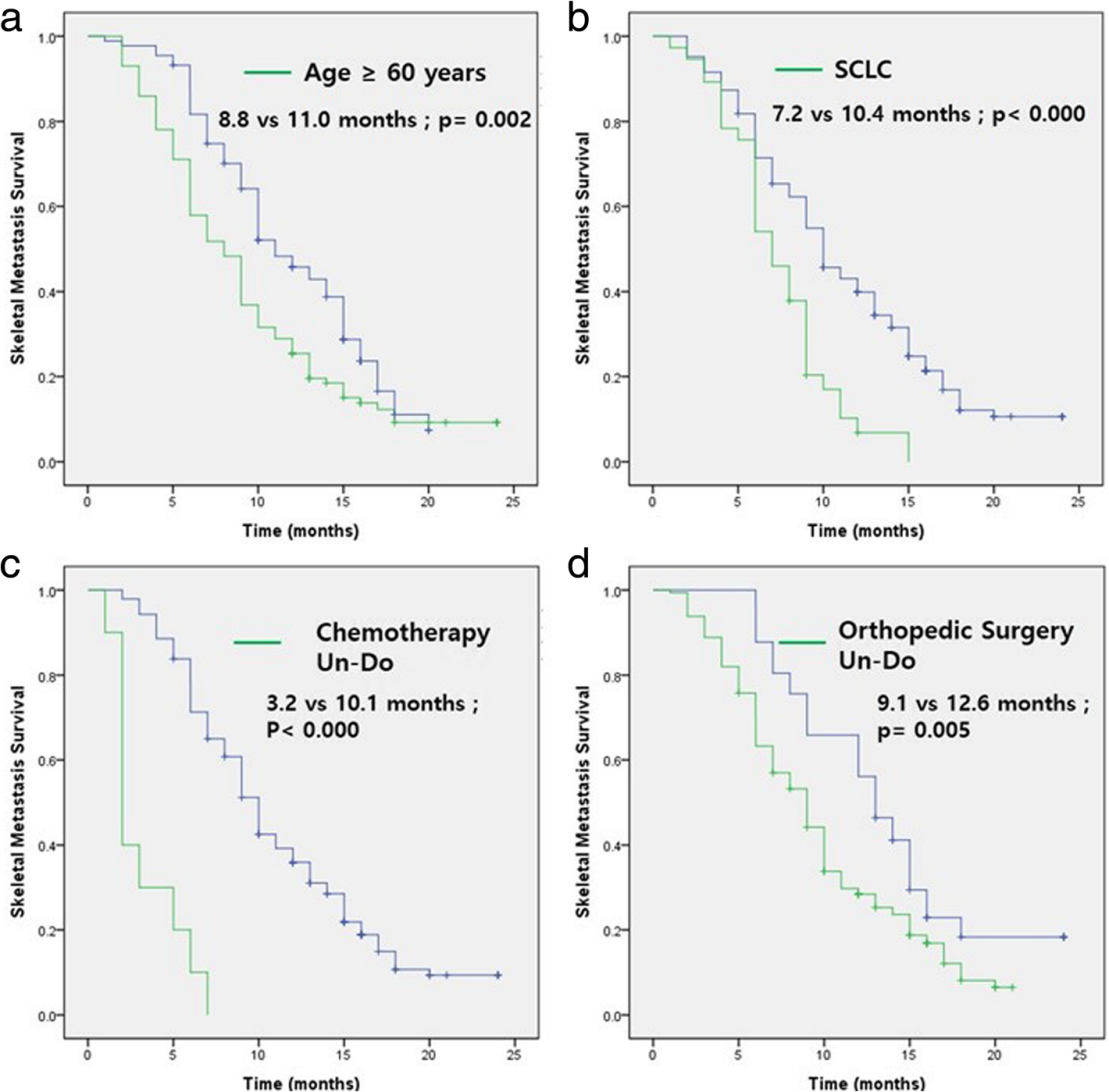
Kaplan-Meier plots of skeletal metastasis survival in **a**) age at diagnosis of lung cancer, **b**) pathologic type of lung cancer, **c**) whether chemotherapy was performed for skeletal metastasis, and **d**) whether orthopedic surgery was performed for skeletal metastasis. Median survivals are expressed in months. Statistical significance was assessed by the log-rank test

The following factors were significantly associated with longer skeletal metastasis survival: lung cancer diagnosed under 60 years of age [HR (95% CI) 1.619 (1.183–2.216), *p* = 0.002], small cell lung cancer pathology type [HR (95% CI) 1.950 (1.309–2.904), *p* < 0.000], chemotherapy for skeletal metastasis [HR (95% CI) 9.155 (4.584–18.284), *p* < 0.000], and orthopedic surgical treatment for skeletal metastasis [HR (95% CI) 1.691 (1.142–2.504), *p* = 0.005] (Fig. 3). In addition, the multivariate analysis performed to determine which factors were an independent prognostic factor revealed that age under 60 years at diagnosis [HR (95% CI) 1.549 (1.124–2.134), *p* = 0.007], small cell lung cancer pathology type [HR (95% CI) 2.045 (1.373–3.047), *p* < 0.000], chemotherapy for skeletal metastasis [HR (95% CI) 7.121 (3.542–14.317), *p* < 0.000], and orthopedic surgical treatment for skeletal metastasis [HR (95% CI) 1.710 (1.148–2.547), *p* = 0.008] were significant independent prognostic factors (Table 3).

**Table 3:** Univariate and multivariate regression analysis of skeletal metastasis survival

Variable	Univariate analysis			Multivariate analysis		
	HR	95% CI	*P*-value	HR	95% CI	*P*-value
Age (< 60 vs. ≥ 60)	1.619	1.183–2.216	0.002	1.549	1.124–2.134	0.007
Sex (male vs. female)	1.157	0.843–1.587	0.338			
Smoking (nonsmoker vs. smoker)	1.189	0.870–1.623	0.141			
Pathology (NSCLC vs. SCLC)	1.950	1.309–2.904	0.000	2.045	1.373–3.047	0.000
EGFR (positive vs. negative)	1.479	0.772–2.833	0.208			
Number of distant organ metastasis (only 1 vs. over 2)	1.058	0.722–1.550	0.247			
Chemotherapy (yes vs. no)	9.155	4.584–18.284	0.000	7.121	3.542–14.317	0.000
Radiation therapy (yes vs. no)	1.291	0.840–1.982	0.059			
Orthopedic surgery (yes vs. no)	1.691	1.142–2.504	0.005	1.710	1.148–2.547	0.008

## Discussion

Majority (~ 50%) of lung cancer patients suffer from bone metastases, mainly spine and rib involvement.

[13, 14]. In a cohort study of 112 patients with lung cancer who had bone metastases, metastatic spinal cord compression (MSCC) occurred in 31cases (27.7%). Patients with MSCC had a 6.1 times greater risk of developing MSCC and poor median survival (4.4 months) and the medial survival time after the occurrence of MSCC was 2.8 months [15]. Possible mechanism of spinal metastasis is access to the vertebral bodies in the thoracic and lumbar spine through the plexus vertebral system and the high bone marrow flow of some skeletal elements [16, 17]. Spine metastasis of lung cancer has been described in several papers, and survival has been relatively well analyzed. Therefore, in this presented article, we discuss pure appendicular skeletal metastasis of lung cancer, except spine metastasis.

The presence of osteolytic lesions leads to weakeness of bone tissue and leads to the emergence of SREs, including hypercalcemia, pathologic fractures, and compression fractures of the spine that might require treatment [18]. Systemic therapies that block osteoclast activity, including bisphosphonates (zoledronic acid) and receptor activator for nuclear factor-κβ ligand (RANKL) inhibitors (denosumab), is known to reduce the incidence of SREs, but they have a modest impact on survival [10]. Denosumab demonstrated superior efficacy than zoledronic acid in delaying the appearance of SREs in NSCLC in a randomized trial; however, denosumab showed no differences regarding osteonecrosis of the jaw, a serious complication encountered in patients treated long-term [19, 20].

Approximately two-thirds of all patients with lung cancer have advanced disease at the time of diagnosis, and these patients are known to have low long-term survival rates. Patients with a lung cancer survival of more than 2 years are reclassified as belonging to the long-term survival group. In long-term survival groups, the survival period can be increased by using different treatment modalities [21–24]. Long-term survivors account for 7.2–12.8% of all patients with advanced lung cancer, and stage and performance status are significant factors in these patients [24–27]. In general, the prognostic factors in metastatic tumors of the skeletal system are performance status, number of skeletal metastases, pain level, and primary carcinoma; in patients with lung cancer, breast cancer, and prostate cancer, these are different from each other [28–33]. In a previous study, Sugiura et al. suggested prognostic factors, including histologic diagnosis, skeletal metastasis location, and use of EGFR-targeted agents, in the prognosis of 118 patients with lung cancer who had skeletal metastasis [13]. In this study, only 71 cases of EGFR mutation were investigated, this is because it is a subject collected from 2005 to 2015. EGFR mutation has been actively investigated since 2011 in South Korea. We investigated the patients with skeletal metastasis. Therefor we do not have control, the patient without skeletal metastasis, to compare whether the EGFR mutation is a risk factor for developing skeletal metastasis. The number of metastatic sites, timing of recurrence, histologic adenocarcinoma, and treatment modality for metastatic tumors are known to be significant prognostic factors for recurrent lung cancer [34–36]. In this study, survival after the diagnosis of skeletal metastasis is discussed, so the results are somewhat different for the factors affecting survival rates.

**Fig. 4:**
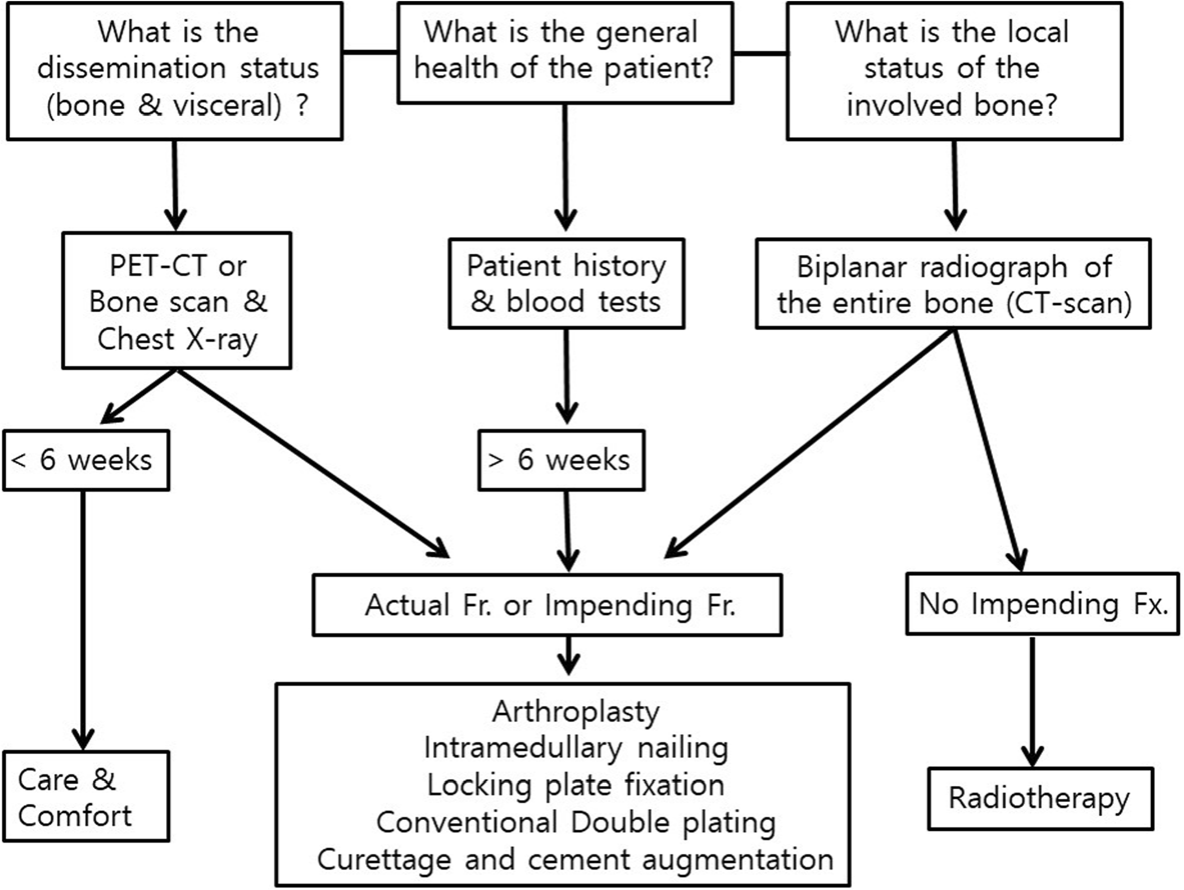
Decision-making diagram for skeletal metastasis in patients with lung cancer

In the treatment principles of pathologic fractures of the long bones, bone dissemination can be grouped into three categories: solitary lesion, oligometastases, or diffuse. Solitary lesions can be treated with curative measures. Especially for patients with kidney and breast cancers, it is hypothesized that more aggressive local treatment might improve survival in the case of oligometastases [37]. However, skeletal metastasis from lung cancer is generally treated palliatively. This tendency is due to the fact that patients with lung cancer have more frequent and more severe pulmonary dysfunction, affecting their ability to tolerate general anesthesia for orthopedic surgery. Moreover, the lifespan in patients with lung cancer is not as long as that in patients with breast or kidney cancers. If orthopedic surgery is planned, comprehensive anesthetic plan should be established including laboratory studies and accurate lung function should be evaluated using high-resolution CT and pulmonary function. A surgical approach to skeletal metastasis is possible if partial anesthesia, including nerve plexus block, is appropriately used. The first step of treatment strategy is to decide whether surgery is required, and this should be determined in a multidisciplinary meeting. Radiotherapy should be considered as standard treatment for small lesions, whereas surgery is useful for actual fractures and impending fractures. The purpose of all treatments is to maintain optimal pain-free function of the extremities and direct weight bearing and mobility. Based on prospective researches, axial cortical involvement of > 30 mm and circumferential cortical involvement of > 50% is considered as risk factors for fracture [38]. The next step is to decide the fixation types. After orthopedic surgery, the expected lifetime and the implant types are important issues. It is fortunate if the implant survives until the patient dies. If the implant wears out before the patient dies, additional decision-making is necessary, including whether to reoperate. The rigid fixation should be durable enough to use during remaining lifetime of the patient, and recovery time should not exceed the life expectancy of the fixation. The aim of our study is to provide the strategy for lung cancer patient with skeletal metastasis. For the patients expected survival is less than 6 weeks, the possible benefits of surgical intervention must be strongly considered and conservative care should be sought. For the cases need absolute requirement for surgical intervention, minimally invasive surgery is needed with short recovery time. For patients with an expected short-term survival (between 6 weeks and 6 months), more invasive procedures are warranted. However, the use of extensive reconstruction or large, complication-prone prostheses should not be considered. For cases with long-term survivor who expected more than 6 months needs comprehensive surgery [39]. Our findings revealed that there was no statistically significant difference in survival after the diagnosis of primary lung cancer between our patient groups. However, survival after diagnosis of skeletal metastasis from lung cancer was significantly longer in patients who underwent orthopedic surgery for skeletal metastasis of lung cancer than in patients who did not receive surgery for skeletal metastasis. Decisions about whether to perform orthopedic surgery for skeletal metastasis were made using the flowchart shown in Fig. 4. Of the 161 patients who did not undergo orthopedic surgery for skeletal metastasis, 9 (5.6%) survived less than 6 weeks; 1 patient (2.4%) had undergone other surgery but died in less than 6 weeks. It may not be possible to exclude the possibility of selection bias from the present study, but this is a limitation of prospective studies. However, it should be understood that this difference was not statistically significant, and we have attempted to comply with standard procedures in decision-making (Fig. 4).

## Conclusion

In our study, we had good prognostic factor for longer survival (less than 60 years old, chemotherapy as treatment for skeletal metastasis, NSCLC rather than SCLC, and underwent orthopedic surgery for skeletal metastasis). Patients died sooner if they had skeletal metastasis at the time of diagnosis of primary lung cancer. Patients with skeletal metastasis from primary lung cancer should be supported in developing a deeper understanding of appropriate treatment to improve their survival and quality of life.

## Abbreviations

CI: Confidence interval
CT: Computed tomography
EGFR: Epidermal growth factor receptor
Fx: Fracture
HR: Hazard ratio
MRI: Magnetic resonance imaging
MSCC: Metastatic spinal cord compression
NSCLC: Non-small cell lung cancer
OR: Odds ratio
PET-CT: Positron emission tomography–computed tomography
SCLC: Small-cell lung cancer
SRE: Skeletal-related event
WBBS: Whole body bone scan
